# Comparing Perioperative Outcomes of Uterine Artery Embolization and Hysterectomy in Insurer and Demographically Diverse Populations: A Retrospective, Multi-Center Database Study

**DOI:** 10.7759/cureus.8653

**Published:** 2020-06-16

**Authors:** Joshua D Lavian, Maziar Sighary, Sean Mooney, Nicole Angel, Neil V Shah, Bassel Diebo, James Walsh

**Affiliations:** 1 Interventional Radiology, State University of New York Downstate Medical Center, Brooklyn, USA; 2 Otolaryngology, State University of New York Downstate Medical Center, Brooklyn, USA; 3 Sarcoma Research, Sarcoma Oncology Center of Southern California, Santa Monica, USA; 4 Orthopaedic Surgery, State University of New York Downstate Medical Center, Brooklyn, USA; 5 Orthopaedics, State University of New York Downstate Medical Center, Brooklyn, USA

**Keywords:** fibroids, leomyoma, uterine artery embolization, uterine fibroid embolization, interventional radiology, hysterectomy

## Abstract

Introduction: Past studies comparing perioperative outcomes of hysterectomy (HYST) and uterine artery embolization (UAE) do not control for demographically and insurer diverse populations. This study sought to identify the 30‑day readmission, 15‑day complication, and minimum 1‑year surveillance reintervention rates of diverse, propensity matched patients undergoing UAE or HYST for uterine leiomyoma.

Methods: Adults from the New York’s Statewide Planning and Research Cooperative System (SPARCS) database 2009‑2013 who underwent HYST or UAE for uterine leiomyoma were retrospectively reviewed and 1:1 propensity matched. Univariate analysis compared demographics, complications, readmissions, and reintervention rates. Binary logistic and linear regression models identified independent predictors of outcomes.

Results: A total of 682 patients were identified, where the number (n) of patients in each cohort was n=341, HYST, and n=341, UAE. Significance levels are shown with p values. No significant differences were identified between HYST and UAE demographics, complication (2.60% HYST vs 2.90% UAE, p=0.816) or readmission rates (3.20% HYST vs 3.80% UAE, p=0.678); 0.30% of UAE patients had a reintervention UAE and 2.90% of UAE patients had reintervention hysterectomy. HYST patients had a significantly longer average length of stay (2.59 days HYST vs 1.63 days UAE, p<0.001). The Deyo-Charlson (Deyo) comorbidity score positively predicted any complication with odds ratio=34.262, 95% confidence interval [4.938, 237.725], and p<0.001, but did not predict readmissions.

Conclusion: HYST patients had significantly longer hospital stays. UAE and HYST had comparable readmission and complication rates. The Deyo comorbidity score was a significant predictor of any complication. This study supports the safety and efficacy of UAE when compared to HYST in demographic and insurer diverse populations.

## Introduction

Uterine fibroids are benign, smooth muscle neoplasms of the uterus. They can cause a variety of symptoms including, but not limited to, menorrhagia, dysmenorrhea, and impaired fertility. Uterine fibroids have an estimated cumulative incidence rate of 70% by age 50 in the United States [1]. In addition to a decrease in quality of life, uterine fibroids account for an estimated $9.4 billion US dollars in annual US health care costs [2].

Hysterectomy (HYST) is an accepted treatment modality for fibroids. Between 1997 and 2005, uterine fibroids were responsible for approximately 30% of all HYSTs among women aged 18-44 [1,3]. However, recent decades witnessed the introduction of alternative treatment methods such as uterine artery embolization (UAE), which was first described in 1995 [4]. Since then, the rate of HYST for uterine fibroids throughout the United States has decreased from 31.4% in 1997 to 26.9% in 2005 while the rate of UAE utilization increased in frequency [1,5].

Numerous single-center studies investigated the effectiveness of UAE and found the procedure to be successful at reducing or eliminating symptoms while also producing minimal complications [6-9]. However, these studies did not compare outcomes to alternative treatment methods. Some of the most frequently cited literature comparing UAE to surgical HYST reported similar findings in terms of UAE outcomes [10-12]. Yet these studies are limited by their demographic information: none of them controlled for race or insurance, if such variables were measured [10-13]. There is well-accepted evidence that patient characteristics, such as form of health insurance, income, and race, may impact patient outcomes in the treatment of uterine fibroids [14]. For example, African American race is a reported risk factor for fibroids [15]. A prospective investigation comparing UAE to HYST at a university teaching hospital, which had a population of 288 patients over a nine-year span, reported zero women of African descent [16]. The authors of that study noted how this may have impacted their UAE complication rates and outcomes [16]. Many well-cited studies do not factor demographics, such as race or insurance, into their analysis [10-12,17,18]. Even when recorded in a study, patients may significantly differ across age, education, and race [19]. Studies such as those funded by the the Netherlands Organization for Health Research and Development are strong pieces of evidence contributing to medical consensus regarding UAE and HYST [11]. Yet, this study and others do not account for demographic information such as the patient’s method of payment, which has been shown to have an impact on the quality of care provided in the United States [20-24]. Based on this gap in the literature, this study sought to identify the 30-day readmission rates, 15-day complication rates, and reintervention rates with minimum 1-year follow-up time of patients undergoing either UAE or HYST for uterine fibroids when successfully propensity matched for demographic information in a multi-center database.

## Materials and methods

Data source

This was a retrospective review of New York’s 2009-2013 Statewide Planning and Research Cooperative System (SPARCS) database. The New York State (NYS) Department of Health receives state and federal funding that is used to maintain the database. All public hospitals in the NYS submit inpatient and outpatient data to SPARCS. This study was deemed exempt by our Institutional Review Board (IRB) as it does not meet the definition of human subject research and only utilizes de-identified health data.

Patient population

The International Classification of Diseases, Ninth Revision, Clinical Modification (ICD9-CM) codes were used to identify patients aged 18 years and over.

The HYST cohort consisted of those with a principal ICD9-CM diagnosis code of leiomyoma (21.89, 21.81, 21.80, 21.82) as well as any one of the following procedural codes: subtotal abdominal hysterectomy (68.39), total abdominal hysterectomy (68.49), vaginal hysterectomy (68.59), laparoscopic supracervical hysterectomy (68.31), laparoscopic total abdominal hysterectomy (68.41), or laparoscopic vaginal hysterectomy (68.51). These specific types of HYST were chosen as they are established in past randomized trials [11]. Codes for radical HYST were not included as the code does not specify the extent of organ/tissue removal beyond the uterus, and it is possible these procedures were more extensive than the average HYST.

The UAE cohort consisted of those with a principal diagnosis of leiomyoma (21.89, 21.81, 21.80, 21.82) as well as any of the following procedural codes for UAE: 68.24, 68.25.

Any patient who had their primary procedure in the last year recorded in the database was eliminated to ensure a minimum one-year surveillance time for reinterventions. Complications and readmissions were monitored for 15 and 30 days, respectively, from the day of the primary procedure (i.e., insurance or lack thereof). A 1:1 propensity score match by age, race, primary method of payment (insurance), Deyo-Charlson (Deyo) comorbidity score, and obesity (defined as body mass index > 30) was conducted prior to comparing operative outcomes of UAE and HYST [25]. The Deyo score and obesity were used specifically because these variables have been shown to impact complication rates of these procedures [10]. The Deyo score was calculated based on the updated assessment by Quan et al. [25].

Exclusion criteria

Patients with any past or present diagnosis of cancer, coagulopathy, uterine prolapse, endometriosis, adenomyosis, or any other vascular procedure were excluded.

Statistical analysis

Propensity score matching was conducted with the PSMATCHING3.03 R plugin for the IBM SPSS (IBM Corp., Armonk, NY). Analyses were conducted with IBM SPSS version 23.0.

Categorical demographic data (race, insurance, obesity) was evaluated with univariate chi-squared analysis. Statistical significance reported is the Pearson chi-square two-sided asymptotic significance. Continuous demographic variables (age, Deyo score, length of stay) were determined to be non-parametric based on Shapiro-Wilk test values of p<0.003. Continuous variables were compared using the Mann-Whitney U test. Complication and readmission rates were calculated using the Pearson chi-square univariate analysis. Bonferroni correction was applied to account for multiple comparisons [26]. The typical significance level, or p-value, of 0.05 was divided by 21, yielding a significance criterion of 0.002 for a complication in the univariate analysis to be considered significant.

Multivariate binary logistic regression models identified independent predictors of having greater than or equal to 1 complication within 15 days and greater than or equal to 1 readmission within 30 days (covariates: age, race, Deyo, obesity, and HYST vs UAE). Linear regression identified independent predictors of hospital length of stay. Regression results were reported as odds ratios (ORs) and confidence intervals (CIs).

## Results

A total of 341 UAE patients met inclusion criteria and were matched to 341 HYST patients. Average age, average Deyo, race, insurance, and obesity status did not differ significantly, indicating a successful propensity match. The results for these variables are shown in Table 1.

**Table 1:** Demographic information for the population studied Demographics include age, Deyo, gender, race, type of insurance used, and obesity status. UAE = uterine artery embolization; HYST = hysterectomy; Deyo = Deyo-Charlson comorbidity score [25].

		HYST	UAE	p value
Sample size		341	341	
		50.00%	50.00%	
Age (years)		44.86	44.85	0.982
Deyo		0.0059	0.0088	0.654
Gender	Male	0.00%	0.00%	
	Female	100.00%	100.00%	
Race	White	24.30%	24.30%	1.000
	Black	47.50%	47.50%	
	Hispanic	10.00%	10.00%	
	Asian or Pacific Islander	3.20%	3.20%	
	Native American	0.30%	0.30%	
	Other	14.70%	14.70%	
Insurance	Medicare	3.50%	3.20%	0.997
	Medicaid	27.00%	27.00%	
	Private insurance	62.80%	62.50%	
	Self-pay	5.00%	5.30%	
	No charge	0.00%	0.00%	
	Other	1.80%	2.10%	
Obese		12.00%	12.00%	1.000

Patients within each cohort had comparable rates of the following complications: puncture (of any surrounding tissue or organs), infection, cardiac, digestive, acute myocardial infarction, acute renal failure, urinary and any complication (indicates the number of patients having at least one complication in that cohort) (HYST=2.60%, UAE=2.90%, p=0.816). Readmission rates did not differ significantly. The HYST cohort had a significantly longer length of stay (HYST=2.59 days, UAE=1.63 days, p<0.001). The UAE cohort had higher rates of reintervention (p values not calculated as probability of UAE or HYST after successful HYST is assumed to be zero); 0.30% of UAE patients had a second UAE and 2.90% of UAE patients had a HYST (both with minimum one-year surveillance time). These results are shown in Table 2.

**Table 2:** Complication, reintervention, length of stay, and 30-day readmission rates for each cohort Fever/chills/malaise/fatigue were post-embolization syndrome characteristics. PVD includes deep vein thrombosis, thrombophlebitis, and other peripheral disease not specified elsewhere in the International Classification of Diseases, Ninth Revision, Clinical Modification. UAE = uterine artery embolization; HYST = hysterectomy; PVD = peripheral vascular disease; MI = myocardial infarction.

	HYST	UAE	p value
Nervous system	0.00%	0.00%	
Cardiac	0.00%	0.60%	0.157
PVD	0.00%	0.00%	
Respiratory	0.00%	0.00%	
Digestive	0.60%	0.90%	0.654
Urinary	0.30%	0.30%	1.000
Vascular	0.00%	0.00%	
Shock	0.00%	0.00%	
Hematoma	0.00%	0.00%	
Puncture	0.30%	0.00%	0.317
Foreign body	0.00%	0.00%	
Infection	1.50%	0.60%	0.254
Fistula	0.00%	0.00%	
Transfusion reaction	0.00%	0.00%	
Fever/chills/malaise/fatigue	0.00%	0.00%	
Acute MI	0.00%	0.30%	0.317
Pneumonia	0.00%	0.00%	
Pneumothorax	0.00%	0.00%	
Lung abscess	0.00%	0.00%	
Acute renal failure	0.00%	0.30%	0.317
Any complication	2.60%	2.90%	0.816
Reintervention with UAE	0.00%	0.30%	
Reintervention with hysterectomy	0.00%	2.90%	
Length of stay (days)	2.59	1.63	<0.001
Any readmission within 30 days	3.20%	3.80%	0.678

Binary logistic regression found Deyo to be a significant predictor of having any complication (OR 34.262, 95% CI [4.938, 237.725], beta coefficient 3.534, p<0.001). Deyo was not a predictor of readmission. Belonging to either the HYST or UAE cohort did not predict readmission or any complication, nor did age, race, or obesity status. The regression results for any complication and readmissions are shown in Table 3.

**Table 3:** Binary logistic regression calculating predictive values of covariates for readmission within 30 days of the primary procedure and for having at least one complication or more within 15 days Complications were as listed in Table 2. For the UAE variable, HYST is the reference variable. UAE = uterine artery embolization; HYST = hysterectomy; OR = odds ratio; Deyo = Deyo-Charlson comorbidity score [25].

	Variable	OR [95% confidence interval]	Beta coefficient	p value
					Readmission within 30 days	UAE	1.181 [0.52, 2.684]	0.166	0.692
Age	0.979 [0.906, 1.057]	-0.022	0.580		
Race	0.837 [0.614, 1.141]	-0.178	0.261		
Deyo	7.067 [0.719, 69.454]	1.955	0.094		
Obesity	1.025 [0.297, 3.541]	0.025	0.968		
Any complication within 15 days	UAE	0.93 [0.366, 2.365]	-0.073	0.879
	Age	0.949 [0.869, 1.036]	-0.053	0.241
	Race	0.951 [0.698, 1.295]	-0.051	0.749
	Deyo	34.262 [4.938, 237.725]	3.534	<0.001
	Obesity	0.65 [0.182, 2.327]	-0.431	0.508

Linear regression found that undergoing HYST was a significant predictor of length of stay (OR -0.964, 95% CI [-1.213, -0.714], beta coefficient -0.278, p<0.001). Age, race, Deyo, and obesity status were not found to be significant predictors of length of stay. The length of stay regression analysis is shown in Table 4.

**Table 4:** Linear regression calculating predictive values of covariates (UAE, age, race, Deyo, obesity) for length of stay For the UAE variable, HYST is the reference variable. UAE = uterine artery embolization; HYST = hysterectomy; OR = odds ratio; Deyo = Deyo-Charlson comorbidity score [25].

	Variable	OR [95% confidence interval]	Beta coefficient	p value
					Length of stay (days)	UAE	-0.964 [-1.213, -0.714]	-0.278	<0.001
Age	-0.011 [-0.034, 0.013]	-0.033	0.376		
Race	-0.054 [-0.132, 0.024]	-0.050	0.175		
Deyo	1.648 [0.181, 3.115]	0.081	0.028		
Obesity	0.292 [-0.093, 0.677]	0.055	0.137		

## Discussion

This study’s findings are in agreement with those of the 2012 Cochrane review (perioperative complication, OR 1.01, 95% CI [0.52, 1.95], 512 women, five trials, I=24%; complications within one month, OR 1.38, 95% CI [0.55, 3.50], one trial, n=121) and other studies indicating there is no significant difference in major complications [13,18,27]. However, a major limitation of this paper is that ICD9-CM codes do not provide enough detail to divide the results of this investigation into major and minor complications. There are mixed findings for the long-term minor complication rates when comparing UAE to HYST. A systematic review by Gupta et al. found the minor complication rate at the five-year follow-up to be significantly higher in the UAE group (OR 2.55, 95% CI [1.26, 5.19], n=144, one trial) [27]. They conducted a complication analysis based on studies such as that by Mara et al., which considered symptoms such as post-operative fever a minor complication [28]. The ICD9-CM coding system is for billing purposes. It may be the case that minor complications that oppose financial incentives, may self-resolve, or those that may go undetected are not recorded, lowering the overall complication rates found in this study. A multi-center retrospective study published after Gupta’s systematic review found UAE to have a lower average five-year complication rate (0.48, 95% CI [0.26, 0.89]) relative to HYST patients [10].

The SPARCS database analysed in this investigation showed that those undergoing UAE have reintervention with minimum one-year surveillance time in the form of a second UAE at a rate of 0.30% and HYST after UAE at a rate of 2.90%. This is in agreement with other studies showing a higher reintervention rate in UAE patients [4,10,13,18,27]. Although not within the scope of this study, it is worth noting secondary hemorrhage as a cause of reintervention after HYST. A retrospective review of 1613 patients found the secondary hemorrhage rate after laparoscopic or abdominal HYST requiring treatment to be 1.3% [29]. While this study calculated a non-significantly higher rate of readmission among the UAE cohort, the 2012 Cochrane review found UAE to have a significantly higher rate of readmissions for minor complications such as post-operative pain [27]. It is possible these numbers will vary based on a hospital’s protocol for time to discharge after a UAE, and that prolonged observation for pain will reduce the number of readmissions for pain.

The findings in this study regarding the length of hospital stay (HYST=2.59, UAE=1.63, p<0.001) reflect those of others, such as Ruuskanen et al. (mean difference of 2.2 days, 95% CI [2.9, 5.38]), indicating a significantly shorter length of stay for UAE patients [13,17,18,27,30]. This dovetails other analyses showing initially reduced or comparable costs of UAE when compared to HYST with accounting for possible reintervention at the five-year mark [18].

As discussed previously, much of the existing literature comparing HYST and UAE for uterine fibroids does not analyse a racially diverse population or match patients for demographics such as race and form of insurance payment despite these traits having an impact on the presentation of fibroids and quality of treatment in the United States [10-24]. This study investigated the revision, readmission, and complication rates in propensity-matched adults undergoing either HYST or UAE for uterine fibroids. After conducting a 1:1 propensity match, demographics (age, race, Deyo, method of payment, obesity status) did not differ significantly. Univariate analysis showed that complication rates and readmission rates between the two cohorts did not differ significantly. Patients undergoing HYST had significantly longer hospital stays. Patients undergoing UAE had a significantly higher chance of reintervention with a second UAE or hysterectomy. The Deyo score was a significant positive predictor of having one or more complications within 15 days, and HYST was a significant positive predictor of having a longer length of stay. The findings in this investigation on racially and insurer diverse cohorts support the existing body of evidence for the safety and efficiency of UAE as an alternative to hysterectomy for uterine fibroids.

Limitations

The database used in this study does not code for the exact position or size of a patient’s uterine fibroids. We attempted to best control for size of fibroids by propensity matching patients for obesity, as there is no variable for body mass index. It does not code for subjective satisfaction with the procedures. The dataset is from the years 2009 to 2013, as we did not have access to newer SPARCS data. Another source of inaccuracy with any database is incorrect original coding of diagnoses and procedures. This study excluded patients with any ICD9-CM vascular procedure other than UAE to limit the influence of incorrect coding.

## Conclusions

Patients propensity matched for demographics undergoing uterine HYST or UAE for uterine leiomyoma have comparable readmission and complication rates. In these racially and insurer diverse cohorts, undergoing HYST was a positive predictor of length of stay. Patients who underwent UAE had higher rates of reintervention. The Deyo comorbidity index was a positive predictor of any complication. Future prospective studies should investigate within UAE cohorts as to whether insurance policy or other demographics have an impact on outcomes.

## References

[1] Merrill RM (2008). Hysterectomy surveillance in the United States, 1997 through 2005. Med Sci Monit.

[2] Cardozo ER, Clark AD, Banks NK, Henne MB, Stegmann BJ, Segars JH (2012). The estimated annual cost of uterine leiomyomata in the United States. Am J Obstet Gynecol.

[3] Viswanathan M, Hartmann K, McKoy N (2007). Management of uterine fibroids: an update of the evidence. Evid Rep Technol Assess (Full Rep).

[4] Ravina JH, Herbreteau D, Ciraru-Vigneron N, Bouret JM, Houdart E, Aymard A, Merland JJ (1995). Arterial embolisation to treat uterine myomata. Lancet.

[5] Goodwin SC, Spies JB, Worthington-Kirsch R, Peterson E, Pron G, Li S, Myers ER (2008). Uterine artery embolization for treatment of leiomyomata: long-term outcomes from the FIBROID registry. Obstet Gynecol.

[6] Salehi M, Jalilian N, Salehi A, Ayazi M (2015). Clinical efficacy and complications of uterine artery embolization in symptomatic uterine fibroids. Glob J Health Sci.

[7] Yoon JK, Han K, Kim MD, Kim GM, Kwon JH, Won JY, Lee DY (2018). Five-year clinical outcomes of uterine artery embolization for symptomatic leiomyomas: an analysis of risk factors for reintervention. Eur J Radiol.

[8] Di Stasi C, Cina A, Rosella F (2018). Uterine fibroid embolization efficacy and safety: 15 years experience in an elevated turnout rate center. Radiol Med.

[9] de Bruijn AM, Adriaansens SJH, Smink M (2019). Uterine artery embolization in women with symptomatic cervical leiomyomata: efficacy and safety. Cardiovasc Intervent Radiol.

[10] Hirst A, Dutton S, Wu O (2008). A multi-centre retrospective cohort study comparing the efficacy, safety and cost-effectiveness of hysterectomy and uterine artery embolisation for the treatment of symptomatic uterine fibroids. The HOPEFUL study. Health Technol Assess.

[11] de Bruijn AM, Ankum WM, Reekers JA, Birnie E, van der Kooij SM, Volkers NA, Hehenkamp WJ (2016). Uterine artery embolization vs hysterectomy in the treatment of symptomatic uterine fibroids: 10-year outcomes from the randomized EMMY trial. Am J Obstet Gynecol.

[12] Jun F, Yamin L, Xinli X, Zhe L, Min Z, Bo Z, Wenli G (2012). Uterine artery embolization versus surgery for symptomatic uterine fibroids: a randomized controlled trial and a meta-analysis of the literature. Arch Gynecol Obstet.

[13] Ruuskanen A, Hippeläinen M, Sipola P, Manninen H (2010). Uterine artery embolisation versus hysterectomy for leiomyomas: primary and 2-year follow-up results of a randomised prospective clinical trial. Eur Radiol.

[14] Borah BJ, Laughlin-Tommaso SK, Myers ER, Yao X, Stewart EA (2016). Association between patient characteristics and treatment procedure among patients with uterine leiomyomas. Obstet Gynecol.

[15] Stewart EA, Cookson CL, Gandolfo RA, Schulze-Rath R (2017). Epidemiology of uterine fibroids: a systematic review. BJOG.

[16] Tropeano G, Amoroso S, Di Stasi C, Di Bidino R, Monterisi A, Petrillo M, Scambia G (2014). Incidence and predictive factors for complications after uterine leiomyoma embolization. Hum Reprod.

[17] Pinto I, Chimeno P, Romo A, Paúl L, Haya J, de la Cal MA, Bajo J (2003). Uterine fibroids: uterine artery embolization versus abdominal hysterectomy for treatment - a prospective, randomized, and controlled clinical trial. Radiology.

[18] Moss JG, Cooper KG, Khaund A (2011). Randomised comparison of uterine artery embolisation (UAE) with surgical treatment in patients with symptomatic uterine fibroids (REST trial): 5-year results. BJOG.

[19] Dutton S, Hirst A, McPherson K, Nicholson T, Maresh M (2007). A UK multicentre retrospective cohort study comparing hysterectomy and uterine artery embolisation for the treatment of symptomatic uterine fibroids (HOPEFUL study): main results on medium-term safety and efficacy. BJOG.

[20] Sohn H (2017). Racial and ethnic disparities in health insurance coverage: dynamics of gaining and losing coverage over the life-course. Popul Res Policy Rev.

[21] Kilbourne AM (2005). Care without coverage: too little, too late [book review]. J Natl Med Assoc.

[22] Safdieh J, Lee YC, Wong A, Lee A, Weiner JP, Schwartz D, Schreiber D (2017). A comparison of outcomes between open hysterectomy and robotic-assisted hysterectomy for endometrial cancer using the national cancer database. Int J Gynecol Cancer.

[23] Amini A, Robin TP, Rusthoven CG (2019). Disparities predict for higher rates of cut-through hysterectomies in locally advanced cervical cancer. Am J Clin Oncol.

[24] Mahal BA, Ziehr DR, Aizer AA (2014). Getting back to equal: the influence of insurance status on racial disparities in the treatment of African American men with high-risk prostate cancer. Urol Oncol.

[25] Quan H, Li B, Couris CM (2011). Updating and validating the Charlson comorbidity index and score for risk adjustment in hospital discharge abstracts using data from 6 countries. Am J Epidemiol.

[26] Armstrong RA (2014). When to use the Bonferroni correction. Ophthalmic Physiol Opt.

[27] Gupta JK, Sinha A, Lumsden MA, Hickey M (2014). Uterine artery embolization for symptomatic uterine fibroids. Cochrane Database Syst Rev.

[28] Mara M, Maskova J, Fucikova Z, Kuzel D, Belsan T, Sosna O (2008). Midterm clinical and first reproductive results of a randomized controlled trial comparing uterine fibroid embolization and myomectomy. Cardiovasc Intervent Radiol.

[29] Paul PG, Prathap T, Kaur H, Shabnam K, Kandhari D, Chopade G (2014). Secondary hemorrhage after total laparoscopic hysterectomy. JSLS.

[30] Nishio J, Iwasaki H, Takagi S, Seo H, Aoki M, Nabeshima K, Naito M (2012). Low-grade central osteosarcoma of the metatarsal bone: a clinicopathological, immunohistochemical, cytogenetic and molecular cytogenetic analysis. Anticancer Res.

